# Supplementary material for: Single-cell mass spectrometry imaging of lipids in HeLa cells via tapping-mode scanning probe electrospray ionization
Source: Commun Chem. 2025 May 14;8:147. doi: 10.1038/s42004-025-01521-2 (PMC12078573; doi:10.1038/s42004-025-01521-2)
Supplement: Supplementary file 1 — Supplementary Information [file 42004_2025_1521_MOESM1_ESM.pdf]

Supplementary Information

Single-Cell Mass Spectrometry Imaging of Lipids in HeLa Cells via Tapping-Mode Scanning Probe Electrospray Ionization

Yoichi Otsuka*[1,2,3], Kazuya Kabayama[2,3,4], Ayane Miura[2], Masatomo Takahashi[5], Kosuke Hata[5], Yoshihiro Izumi[5], Takeshi Bamba[5], Koichi Fukase[2,3,4], Michisato Toyoda[1,2,3]

[1]Department of Physics, Graduate School of Science, The University of Osaka, Toyonaka, Osaka, Japan
[2]Department of Chemistry, Graduate School of Science, The University of Osaka, Toyonaka, Osaka, Japan
[3]Forefront Research Center, Graduate School of Science, The University of Osaka, Toyonaka, Osaka, Japan
[4]Interdisciplinary Research Center for Radiation Sciences, Institute for Radiation Sciences, The University of Osaka, Toyonaka, Osaka, Japan
[5]Division of Metabolomics, Medical Research Center for High Depth Omics, Medical Institute of Bioregulation, Kyushu University, Fukuoka, Fukuoka, Japan

***Corresponding author**
Yoichi Otsuka
Email: otsuka@phys.sci.osaka-u.ac.jp
Telephone: +81-6-6850-5748

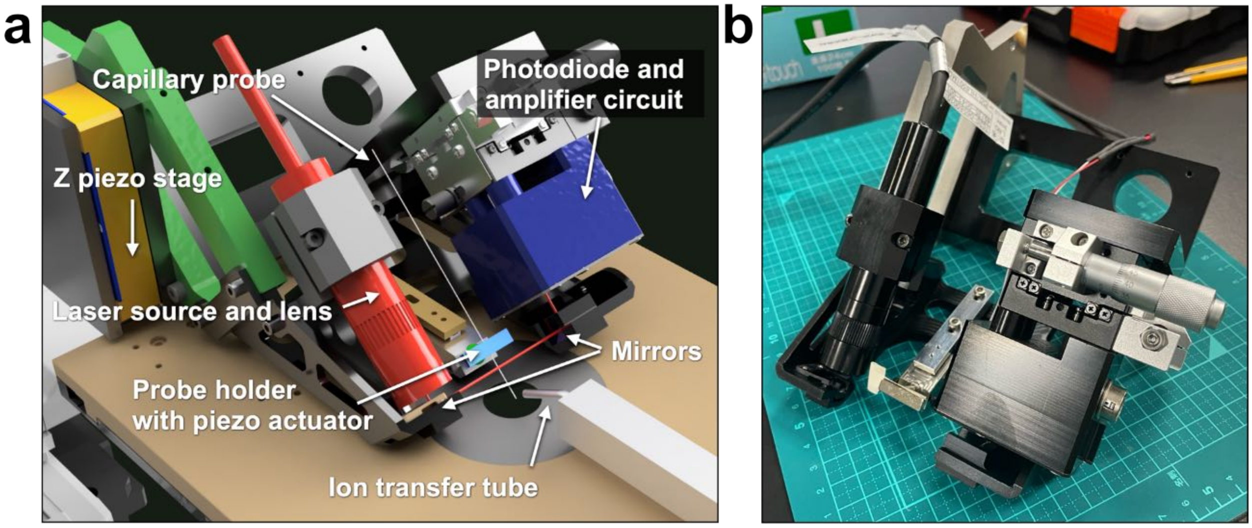

Supplementary Fig. 1. Overview of the new t-SPESI unit for use with an inverted fluorescence microscope.

(**a**) Rendered image of a 3D model, showing the name of each component of the t-SPESI unit indicated by arrows. Each component is color coded for identification. (**b**) Photograph of the t-SPESI unit.

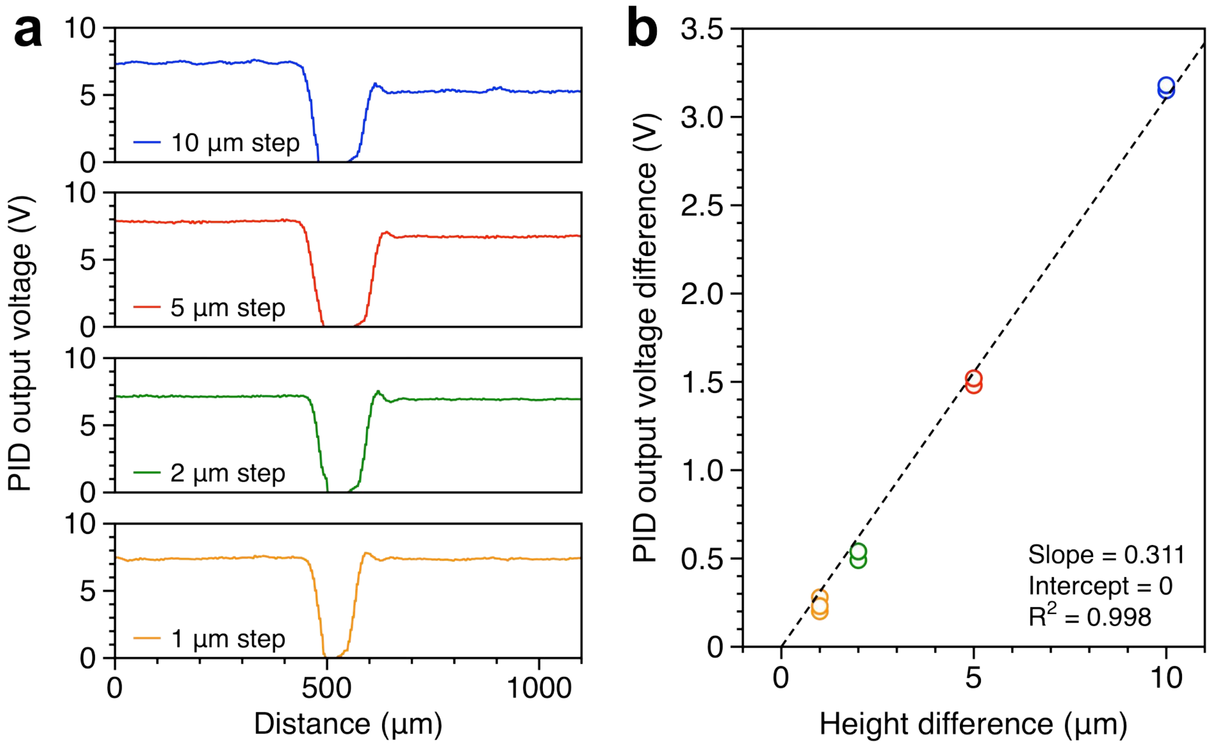

Supplementary Fig. 2. Relationship between the sample height and feedback controller output. (**a**) Section profiles showing the relationship between the output signal from the feedback control circuit (PID output voltage) and the probe position when the probe scans between samples of different heights. (**b**) Relationship between the height difference of the samples and the PID output voltage difference. The linear fit result is shown as a dashed line. The inset shows the slope and R^2 value of the dashed line. It was confirmed that the calibration data could be linearly fitted. In addition, the position resolution of the piezo stage for controlling the height of the probe was 1 nm, and the repeat positioning accuracy was ±1 nm; therefore, the height of the sample in the range of up to 10 µm could be evaluated with nanoscale accuracy.

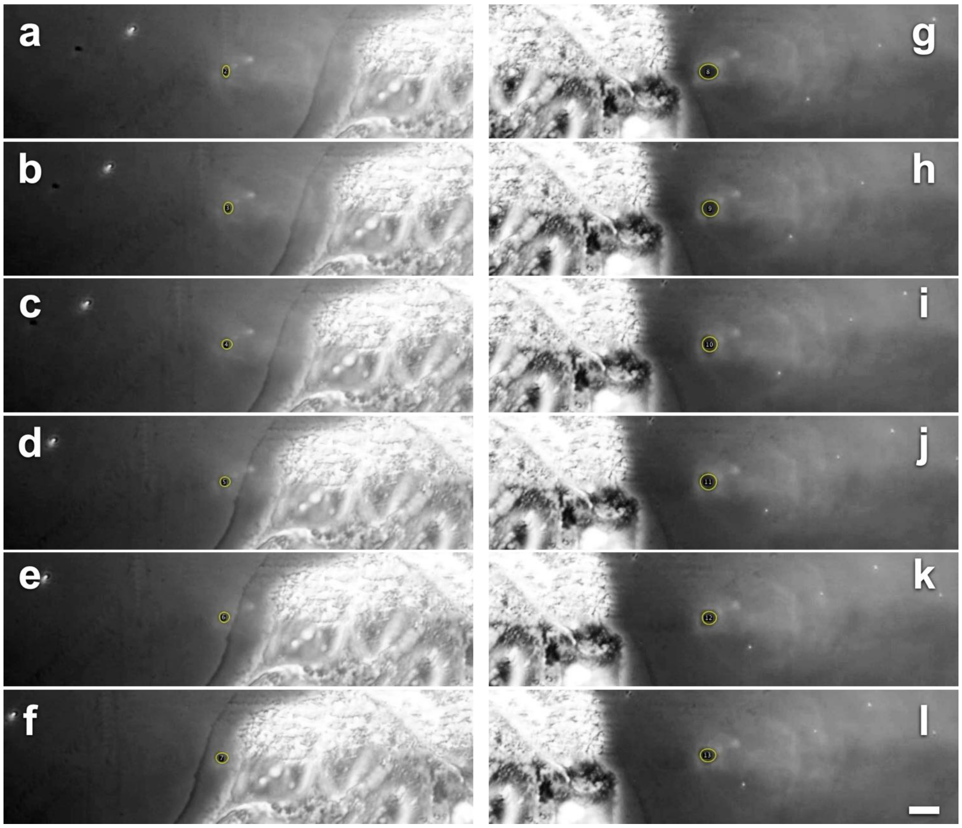

Supplementary Fig. 3. Evaluation of spreading of the liquid bridge on the glass substrate during a single-line scan of the probe.

(**a**) and (**l**) correspond to the snapshots at the beginning and end of the probe scan, respectively, and the others are snapshots taken during the scan, arranged in order of increasing time. The area of the liquid bridge is indicated by the yellow circles. Scale bar: 10 μm.

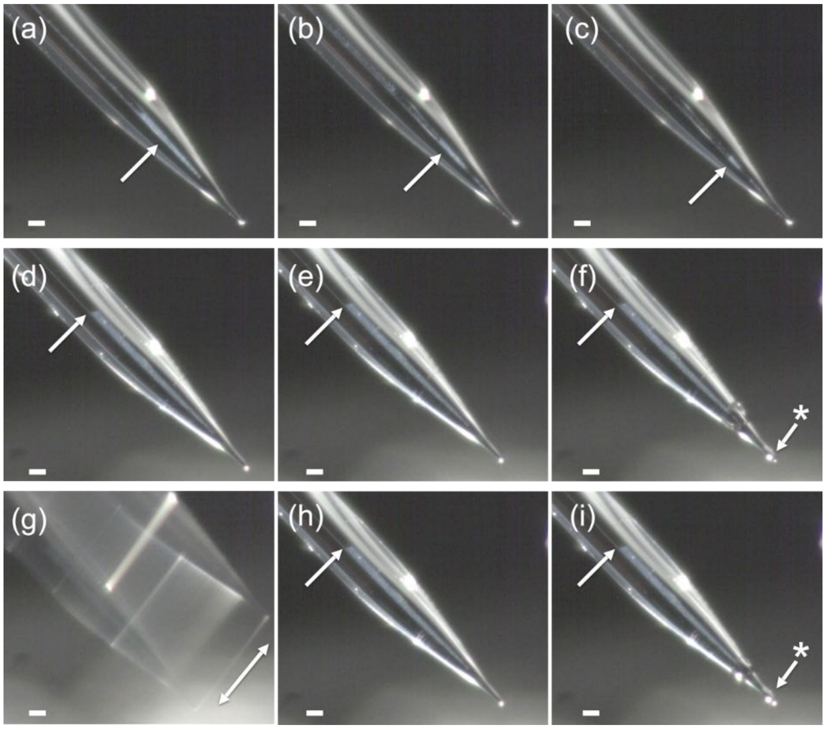

Supplementary Fig. 4. Comparison of the movements of octadecylsilyl-group-modified and amino-group-modified particles in a capillary probe.

(**a**-**c**) Observation results when +4 kV was applied to the solvent using a probe filled with octadecylsilyl-group-modified particles (O-particles). (**a**), (**b**), and (**c**) Images before applying the voltage, 45 s after applying the voltage, and 100 s after applying the voltage. During voltage application, the particles moved upward with time, and the number of particles at the tip of the probe (arrows in (**a**), (**b**), and (**c**)) decreased.

(**d**-**f**) Observation results when +4 kV was applied to the solvent using a probe filled with amino-group-modified particles (N-particles). (**d**) and (**e**) Images immediately after and 150 s after voltage application, respectively. The particles in the probe (arrows in (**d**) and (**e**)) did not move with time and remained at the same position. In addition, when the voltage was set to 0 V, the particles in the probe did not move with time and remained at the same position (arrows in (**f**)). The solvent was confirmed to flow out of the tip of the probe (asterisk in (**f**)).

Next, with a voltage of +4 kV applied to the solvent, the probe was oscillated in the vertical direction for 110 s at a resonant frequency of 523 Hz. The arrows in (**g**) indicate the oscillation direction. When the oscillation stopped, the particles in the probe were observed to be present at the same position (arrows in (**h**)). Furthermore, even when the voltage was set to 0 V, the particles in the probe did not move with time and remained at the same position (arrow in (**i**)). In addition, the solvent flowed out of the tip of the probe (asterisk in (**i**)). Scale bar: 100 μm.

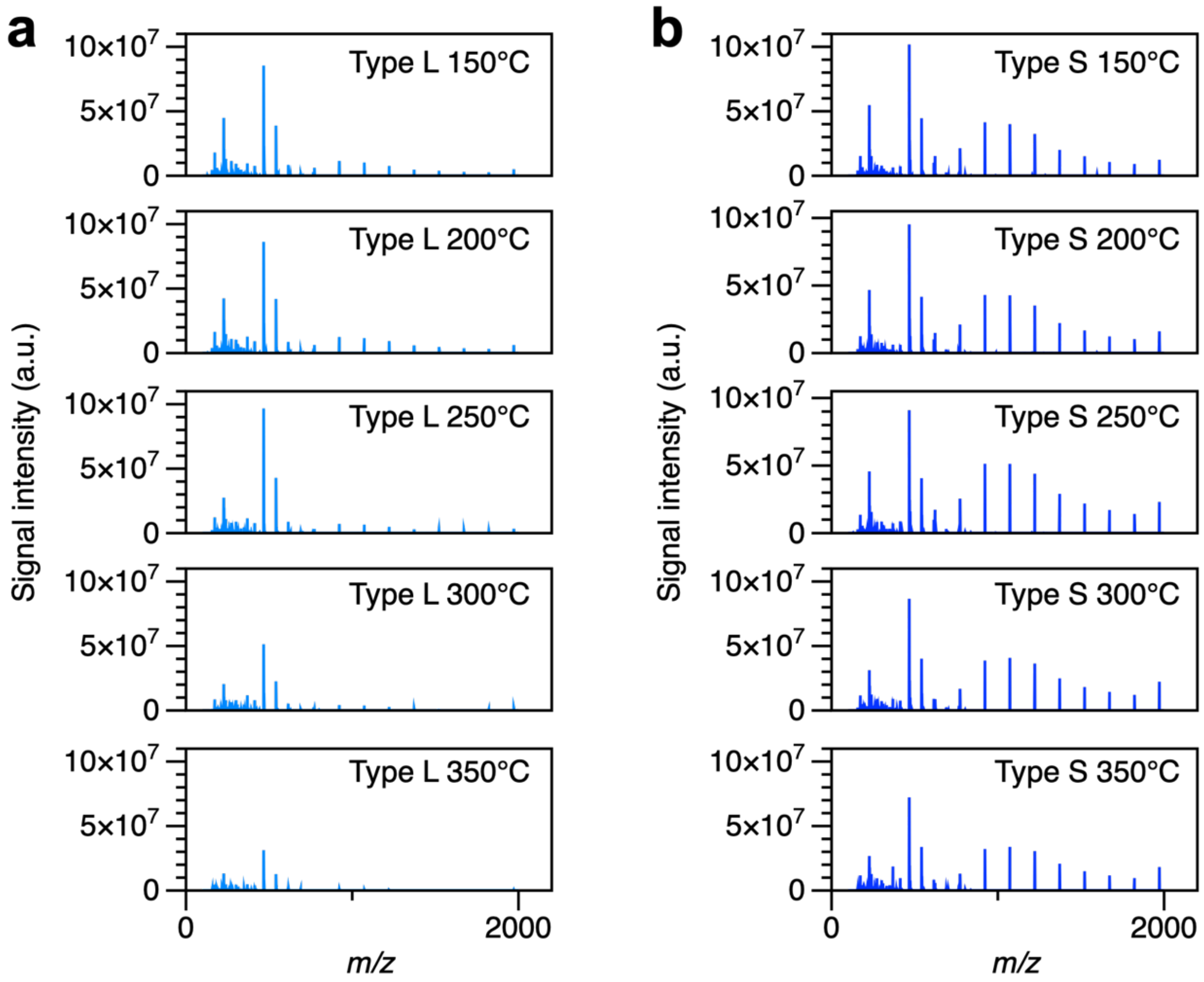

Supplementary Fig. 5. Comparison of mass spectra of NaI cluster ions obtained with different ion transfer tubes

(**a**) and (**b**) Mass spectra of the NaI cluster ions generated by ESI using different ion transfer tubes (types L and S, respectively). The temperature of the ion transfer tube was varied in the range of 150–350°C.

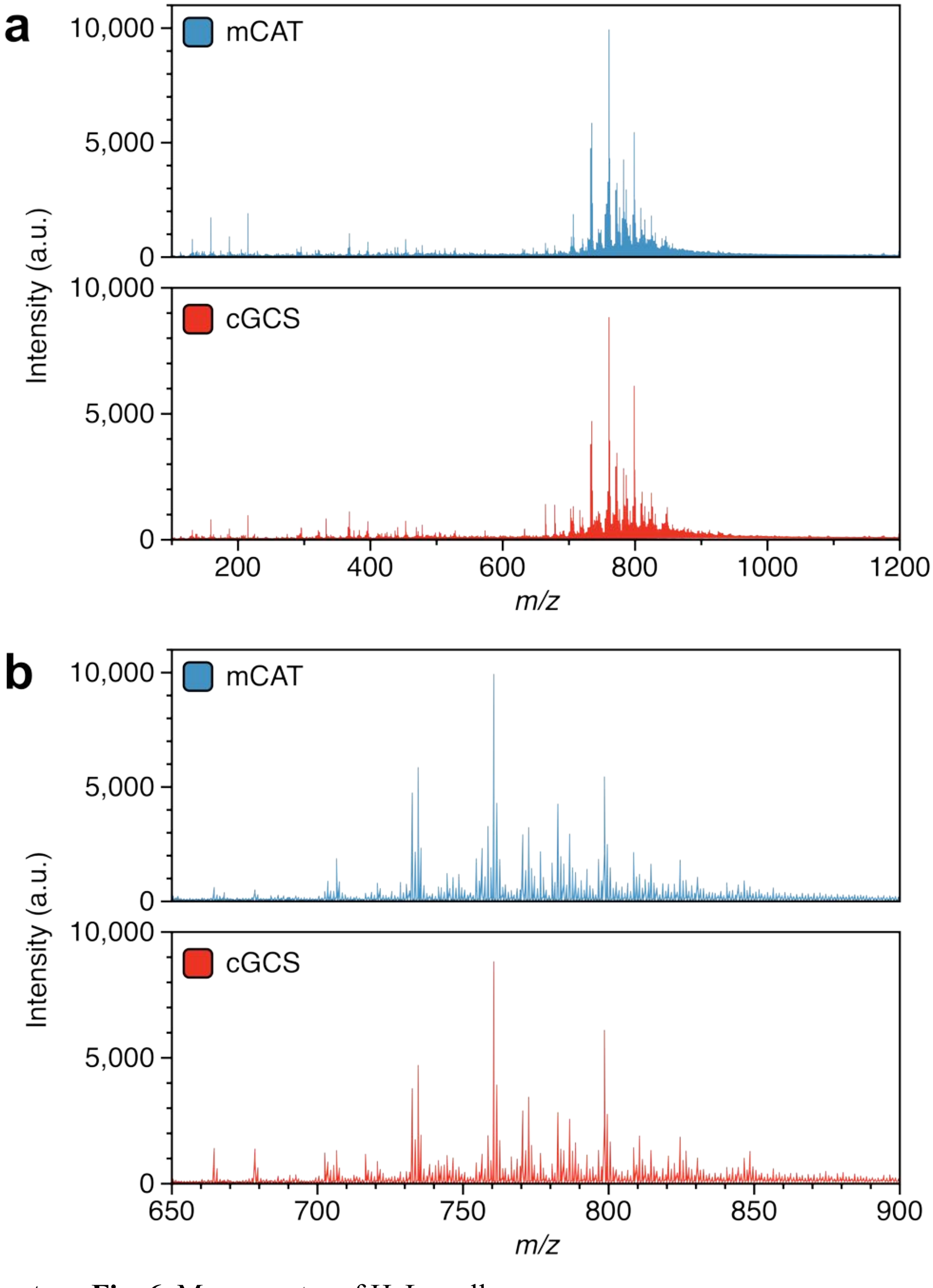

Supplementary Fig. 6. Mass spectra of HeLa cells.
Average mass spectra of mCAT and cGCS cells. (**a**) Mass spectra in the range of m/z 100–1200, and (**b**) enlarged mass spectra in the range of *m/z* 650–900.

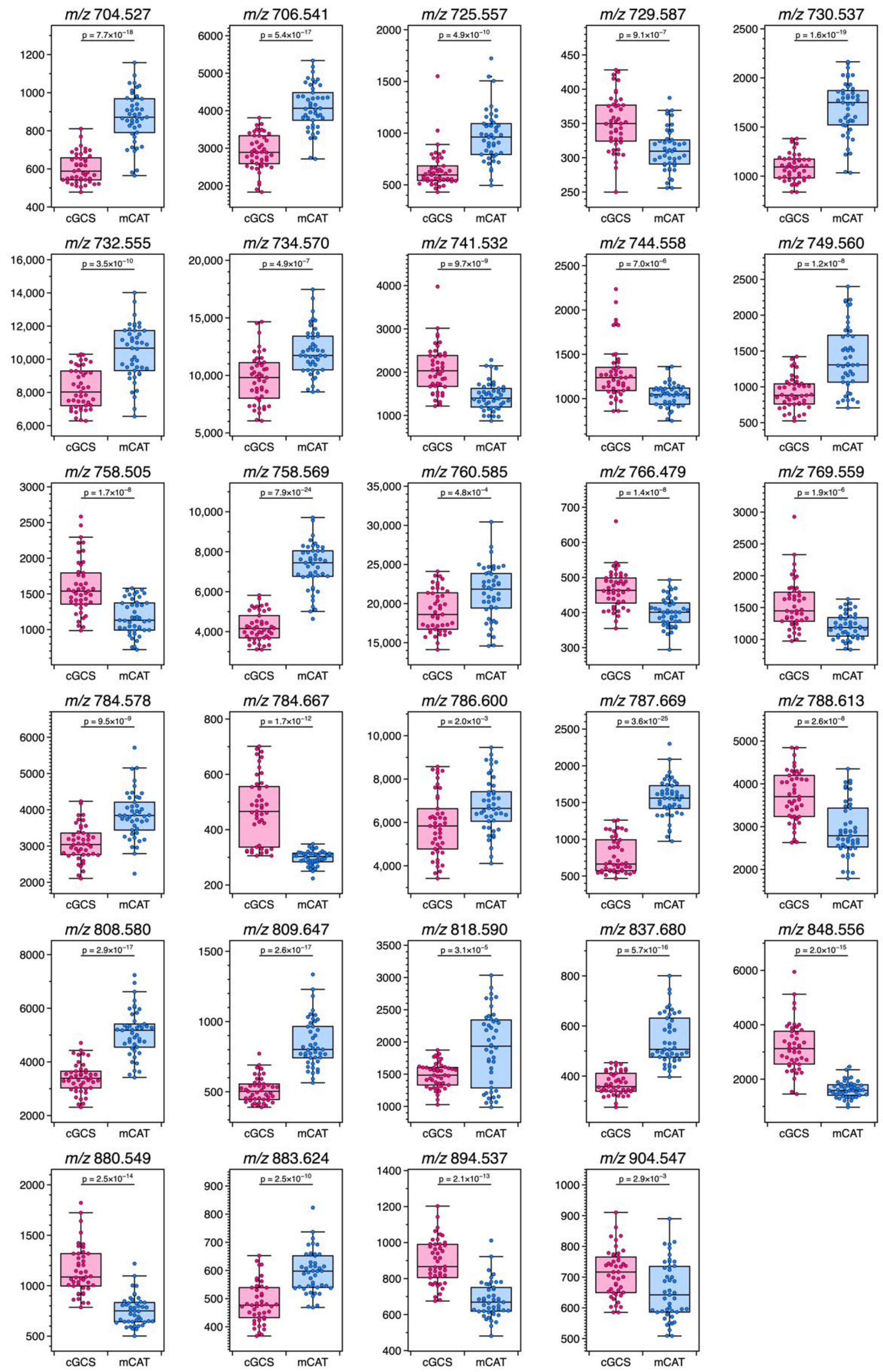

Supplementary Fig. 7. Comparison of lipid ion signal intensities between cell types.

mCAT: Region 1

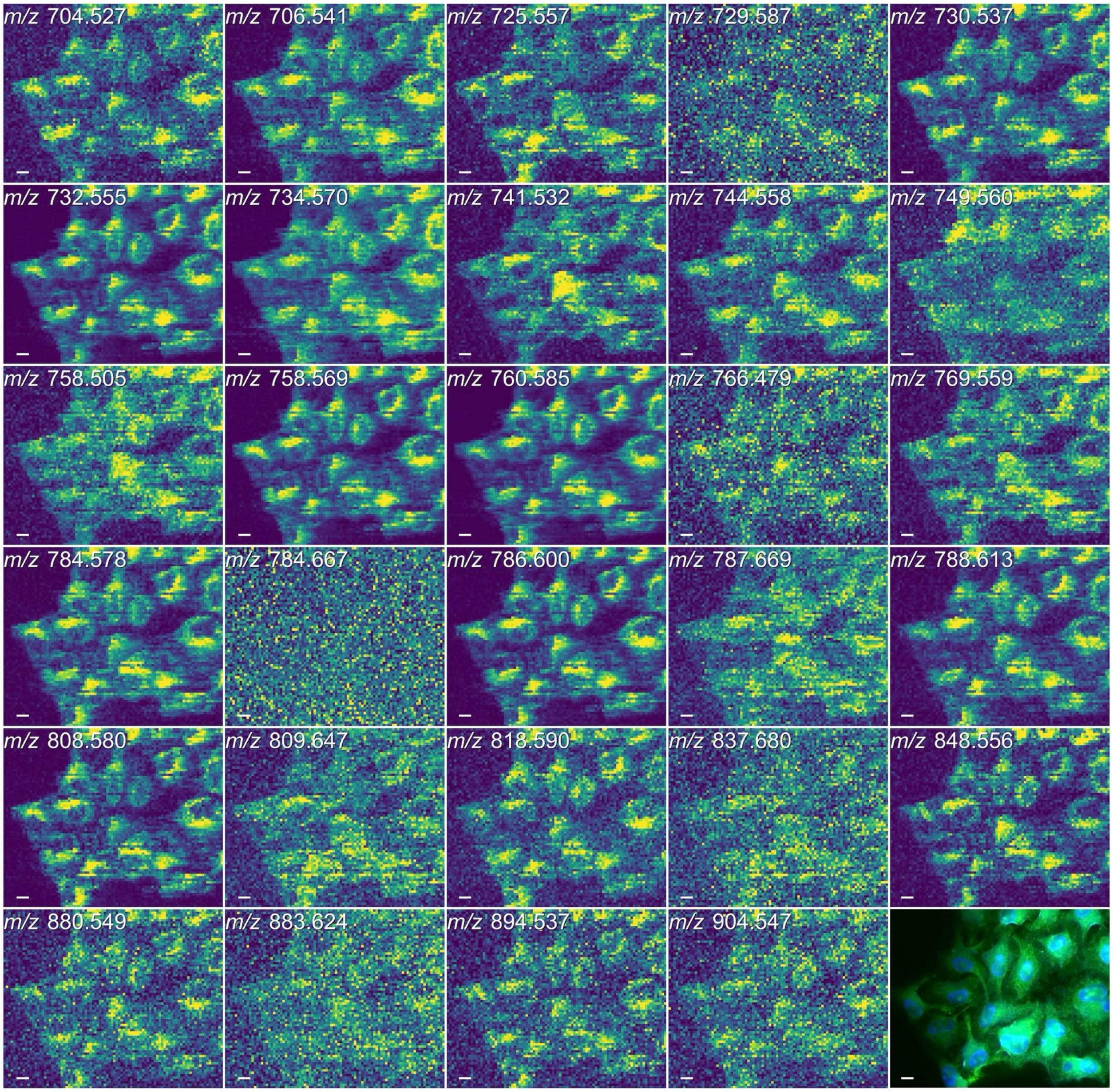

mCAT: Region 2

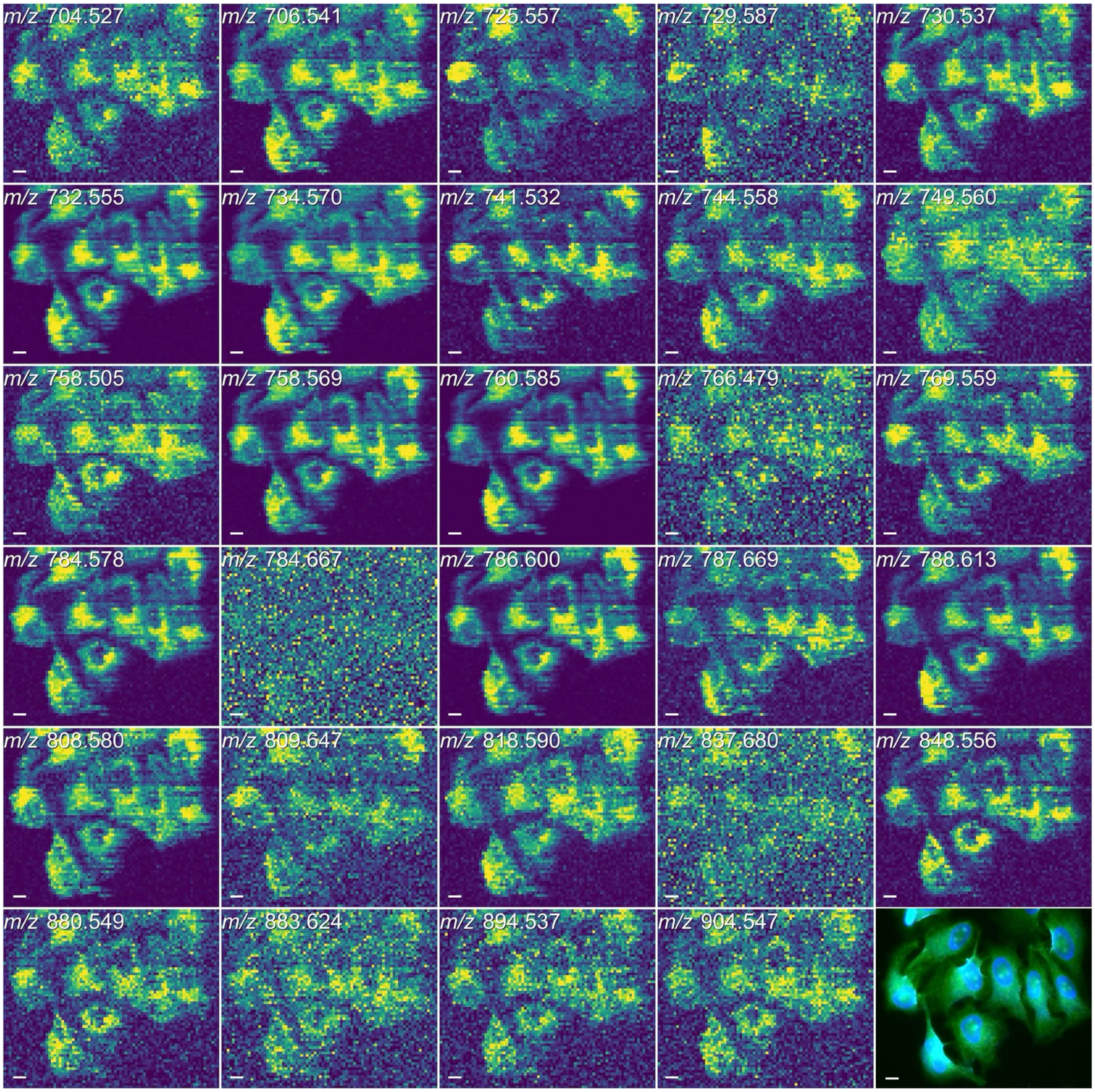

mCAT: Region 3

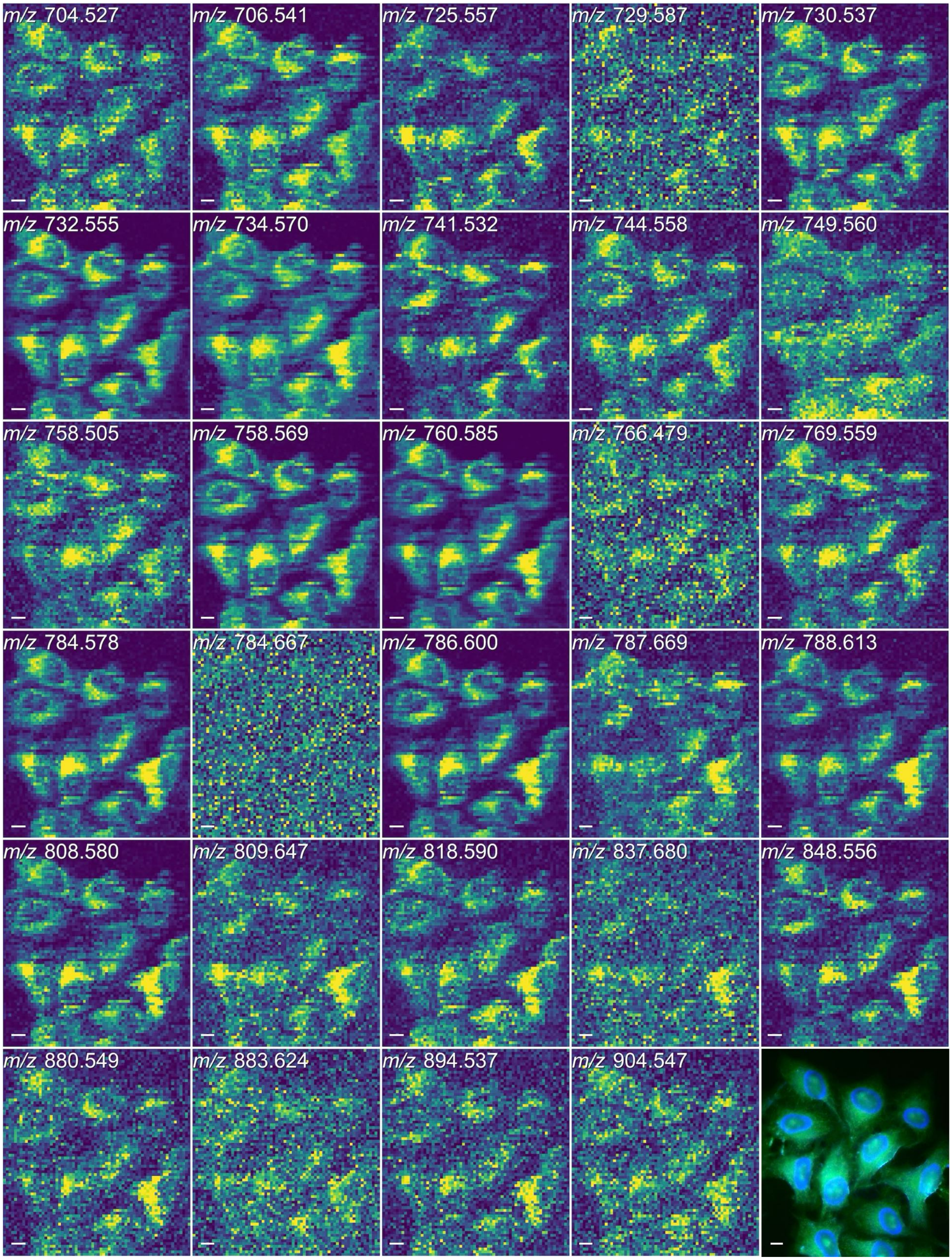

mCAT: Region 4

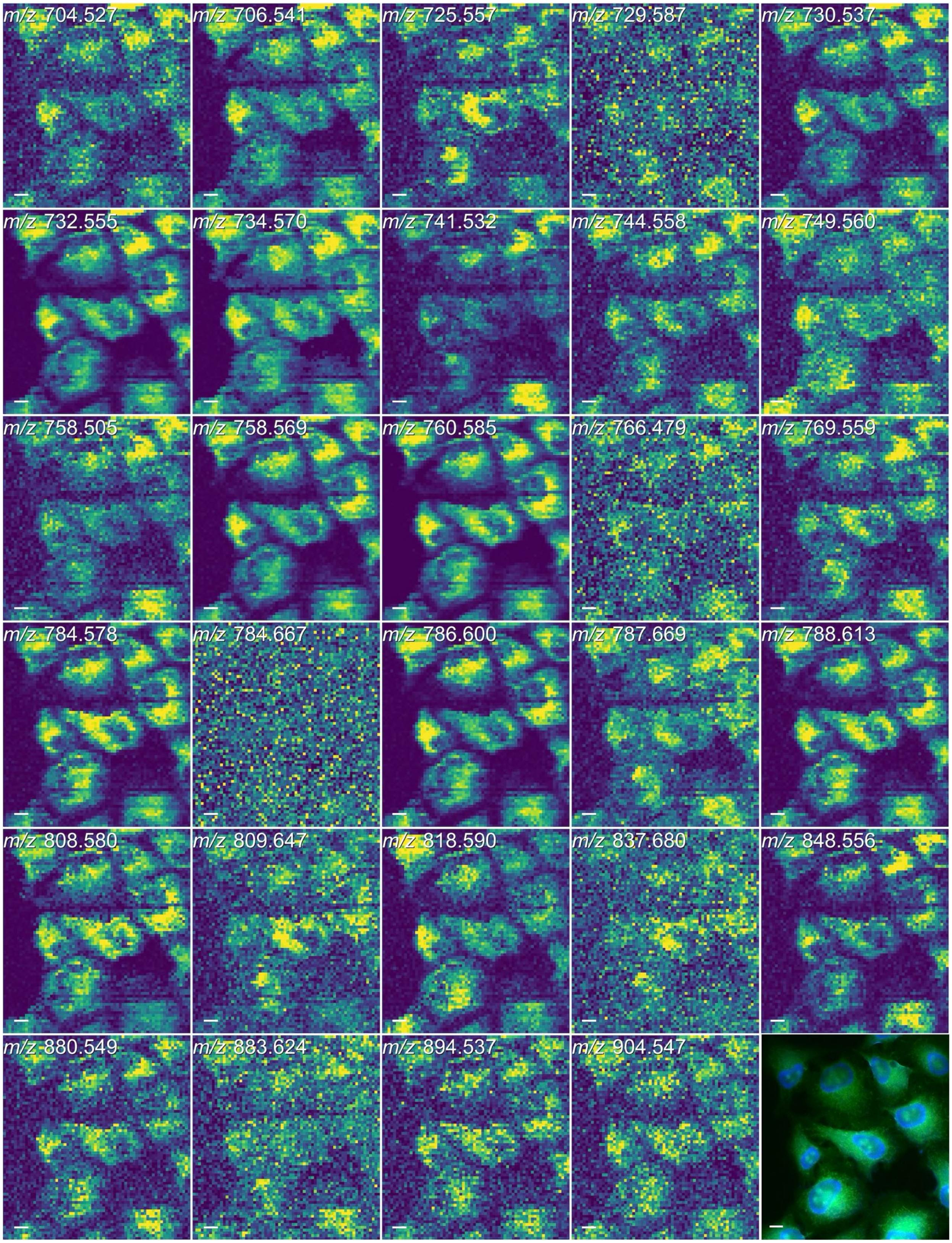

mCAT: Region 5

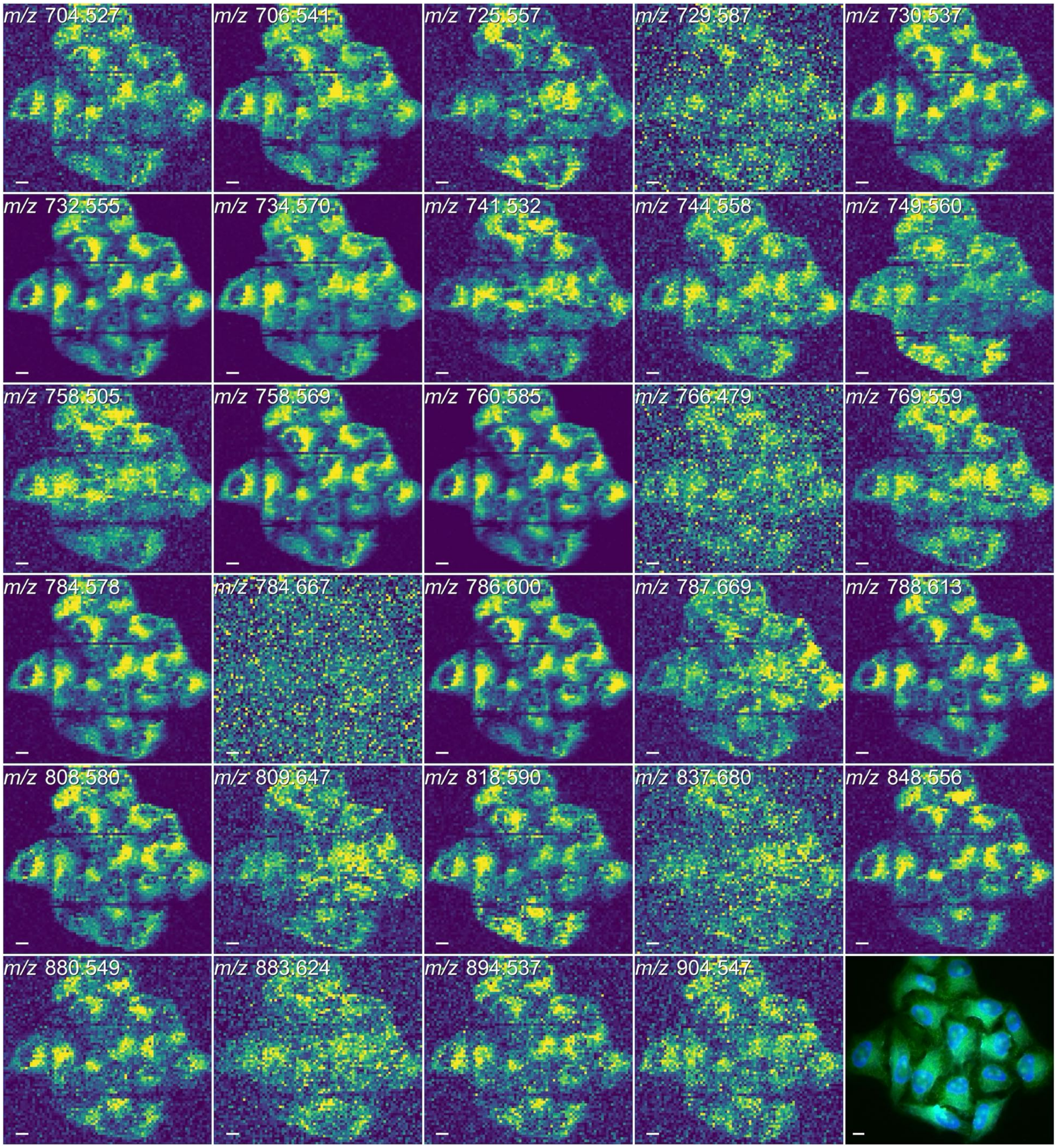

cGCS: Region 1

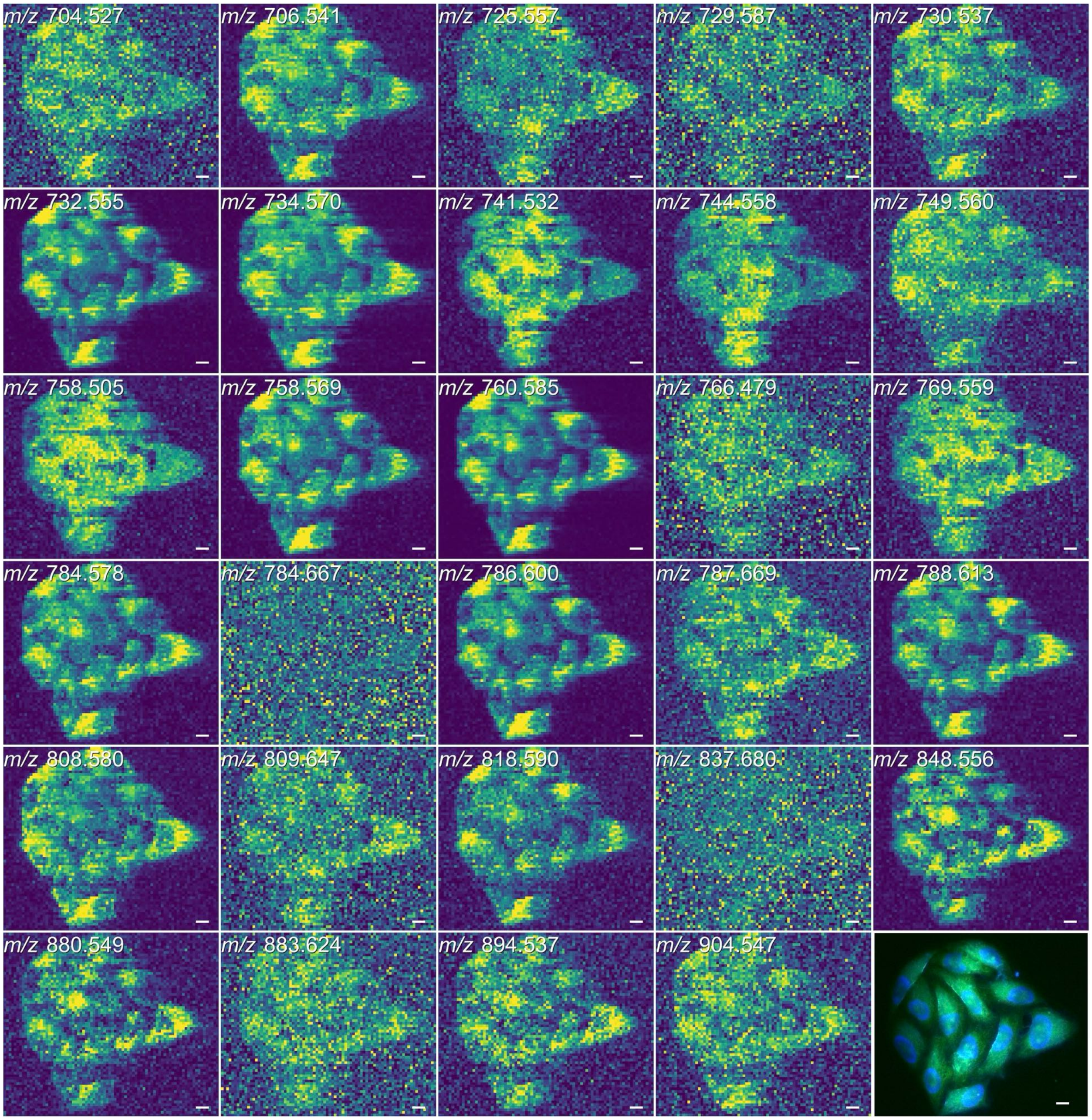

cGCS: Region 2

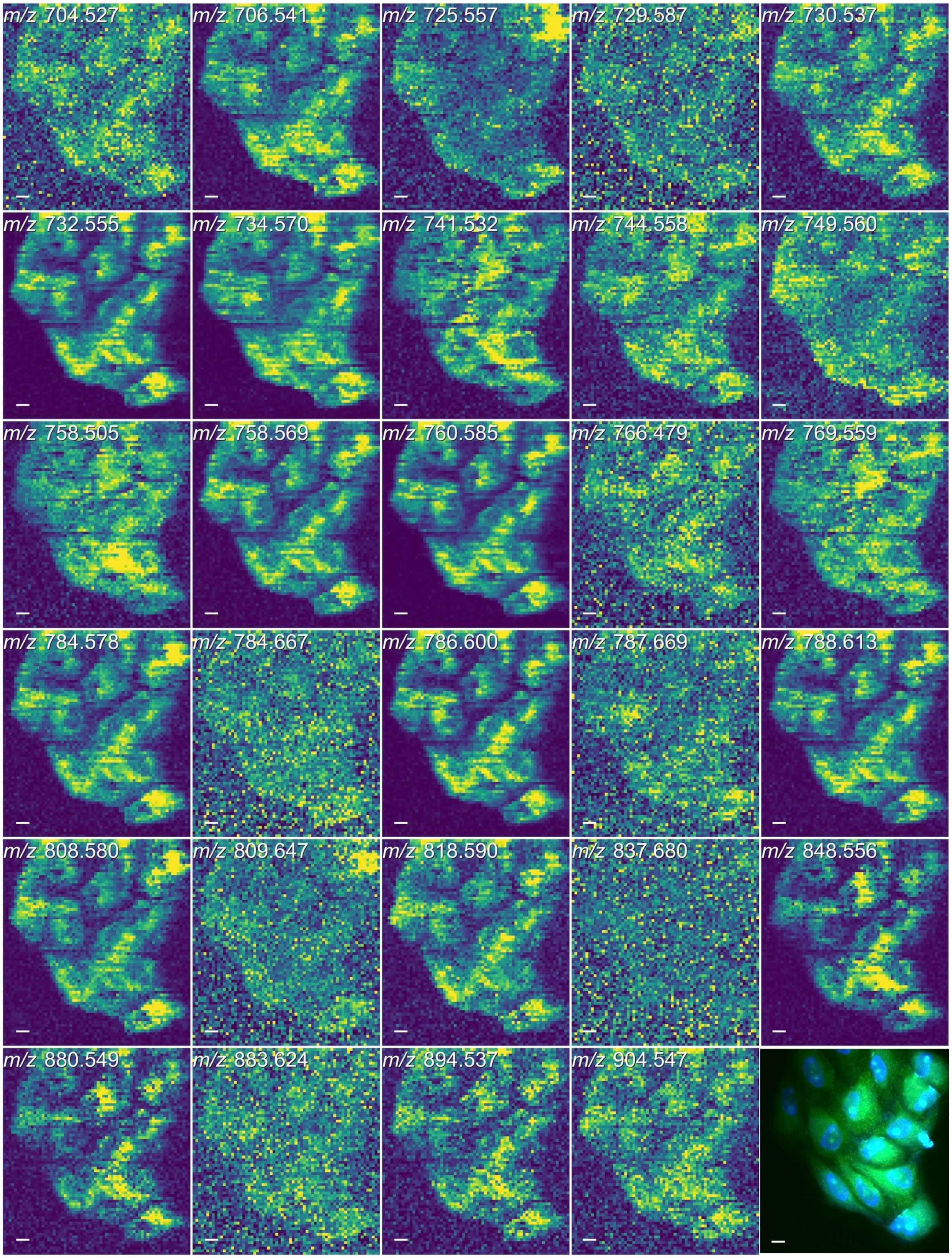

cGCS: Region 3

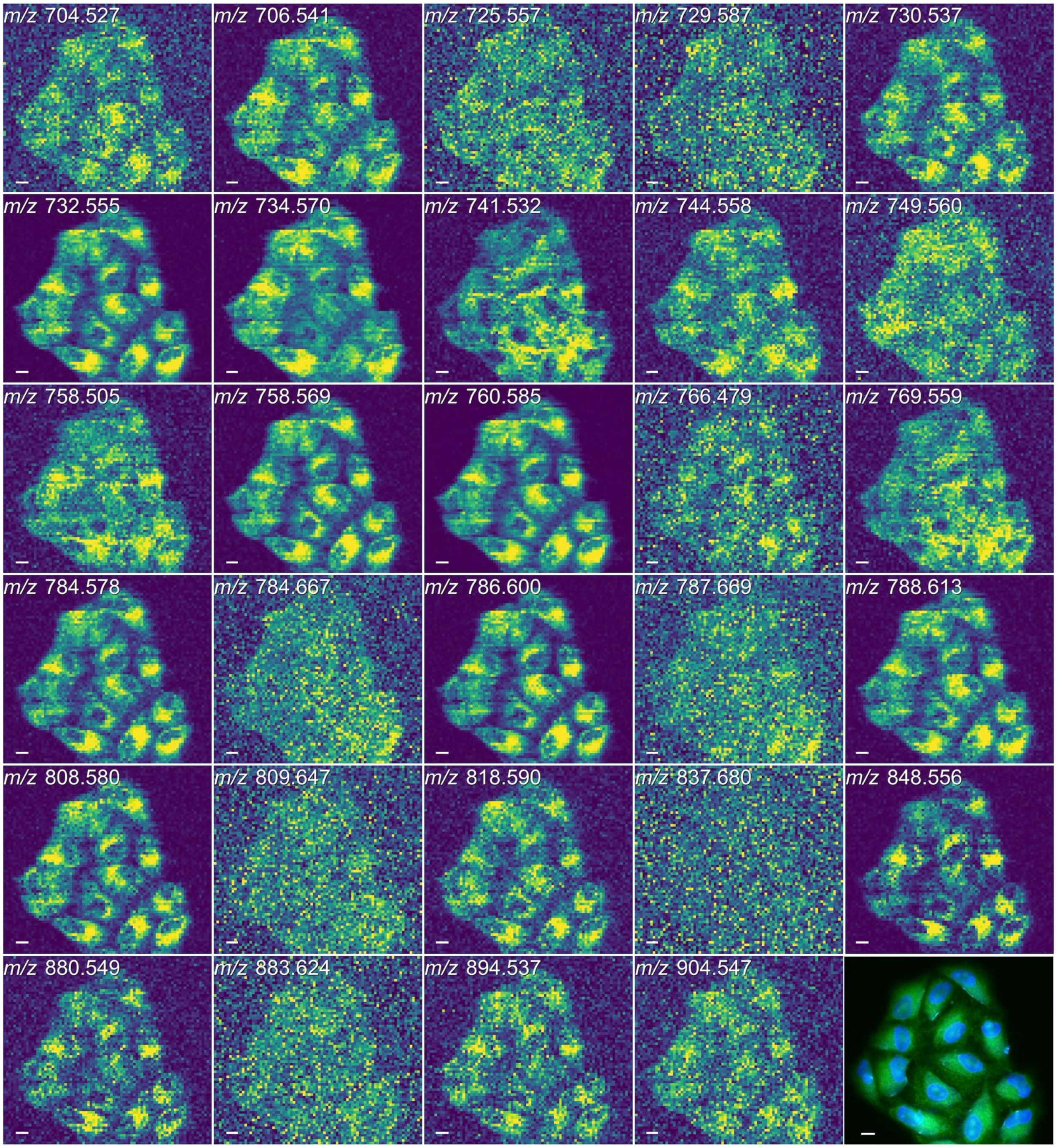

cGCS: Region 4

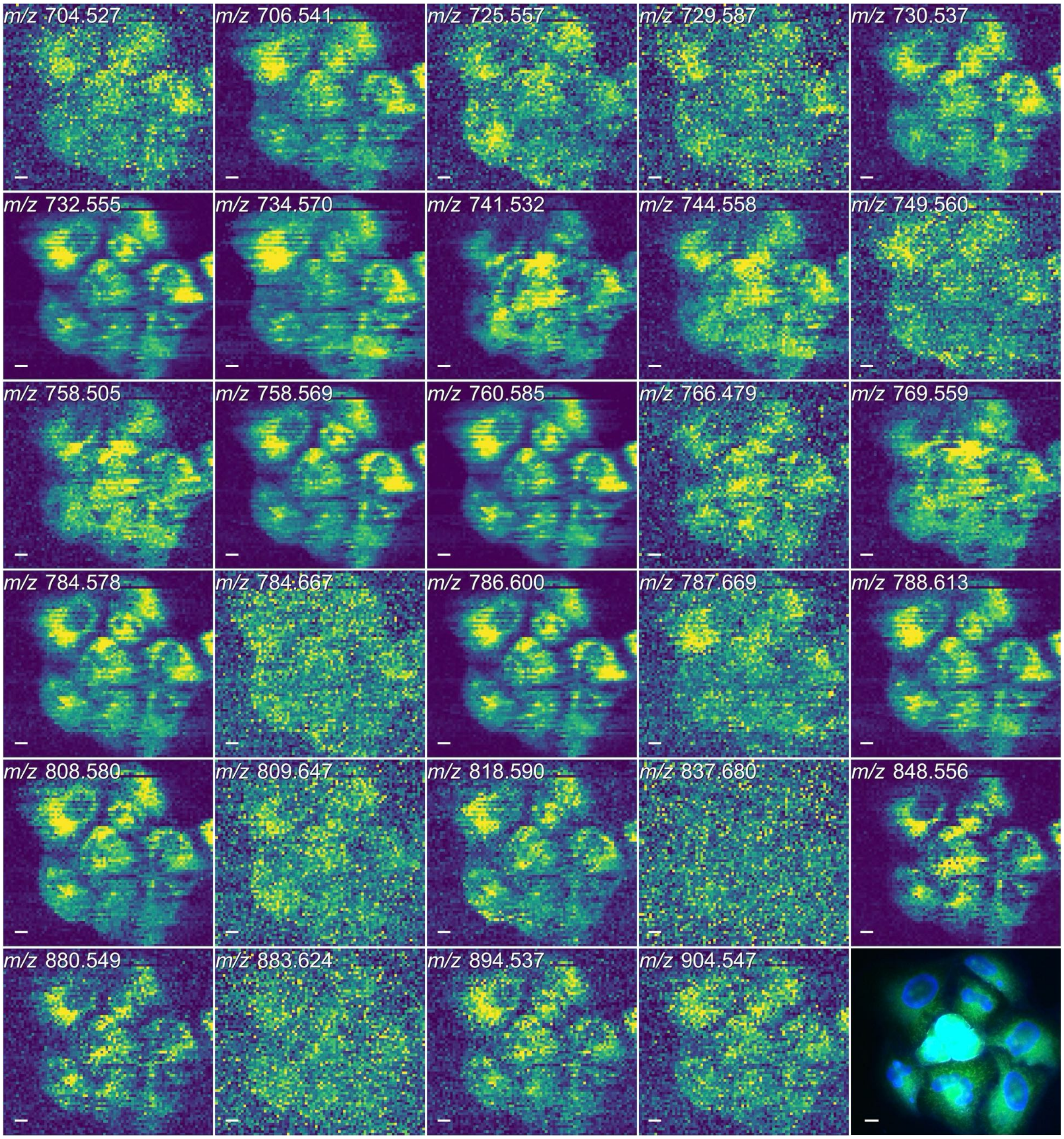

Supplementary Fig. 8. Ion images of HeLa cells obtained by t-SPESI-MSI. Scale bar: 10 μm.

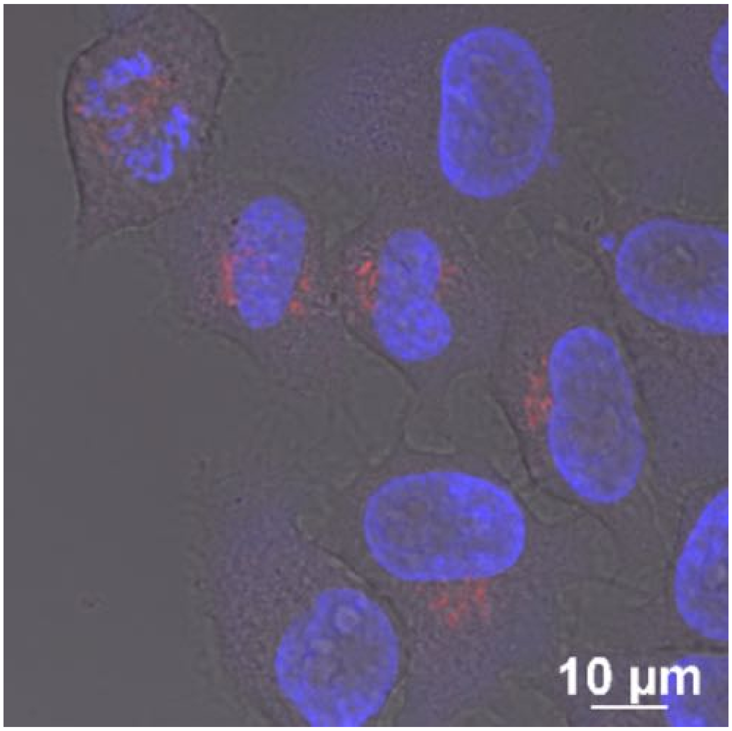

Supplementary Fig. 9. Observation of the nucleus and Golgi apparatus in HeLa cells.

To visualize the nucleus and Golgi apparatus in HeLa mCAT cells, the nucleus was stained with Hoechst 33342 (blue region), and red fluorescent protein for binding to N-acetylgalactosaminyltransferase in the Golgi apparatus was expressed. To show the shape of the cell, a fluorescence image was overlaid on the bright-field image.

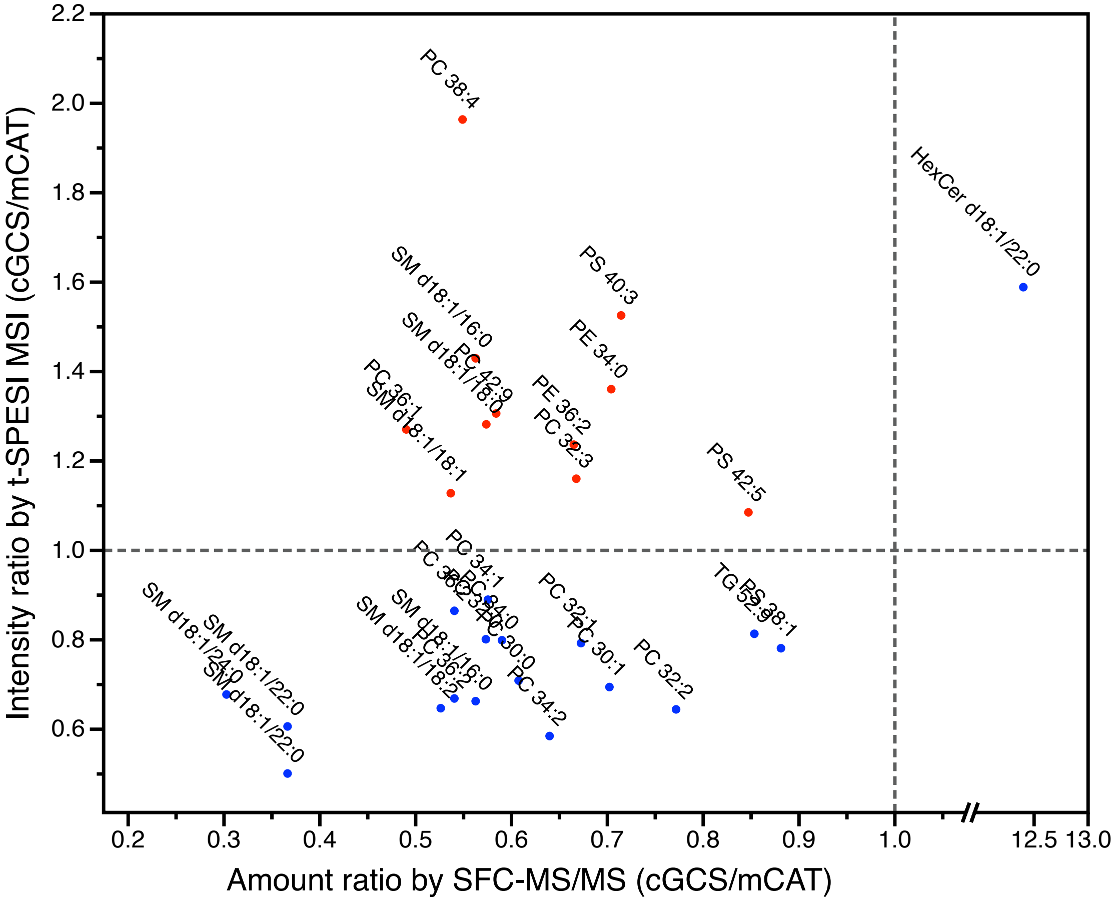

Supplementary Fig. 10. Comparison of lipid ion signal intensity ratios acquired via t-SPESI MSI and lipid amount ratios measured via SFC-MS/MS.

The horizontal axis shows the ratio of the amounts of lipids in cGCS and mCAT cells obtained via SFC-MS/MS. The vertical axis shows the ratio of the signal intensities of lipid ions in cGCS and mCAT cells obtained via t-SPESI MSI. The numerical values for each ratio are listed in Supplementary Table 5. Each data point is labeled with a lipid species. The blue dots indicate that the ratios determined via SFC-MS/MS and t-SPESI MSI are positively correlated, and the red dots indicate that they are negatively correlated.

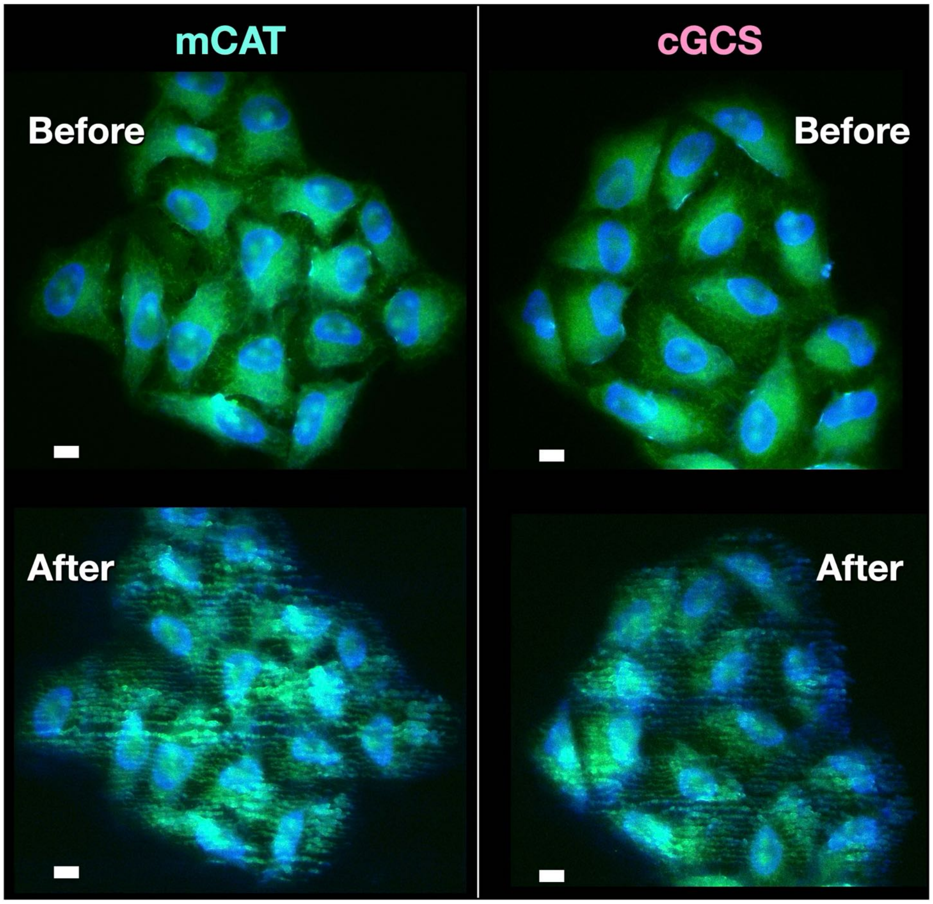

Supplementary Fig. 11. Comparison of fluorescence microscope images of HeLa cells before and after single-cell imaging. Scale bar: 10 μm.

Supplementary Table 1. Analysis results for spreading area of liquid bridge. The types of images correspond to those shown in Fig. 3.

Image name	Area (μm^2)	Diameter (μm)
a	6.5	2.9
b	8.4	3.3
c	7.8	3.2
d	7.8	3.2
e	8.4	3.3
f	10.1	3.6
g	22.0	5.3
h	20.6	5.1
i	18.1	4.8
j	20.6	5.1
k	14.9	4.4
l	16.1	4.5

Supplementary Table 2. Parameters of P-2000 for the fabrication of capillary probes.

	HEAT	FIL	VEL	DEL	PUL
LINE 1	235	0	15	225	0
LINE 2	235	0	15	225	0
LINE 3	235	0	15	225	0
LINE 4	235	0	15	225	0
LINE 5	235	0	10	225	0
LINE 6	235	0	10	225	0
LINE 7	235	0	10	225	0

Supplementary Table 3. List of NaI cluster ions in positive ion mode mass spectra.

m/z	Ion
172.884	$(NaI)Na^+$
322.778	$(NaI)_2Na^+$
472.673	$(NaI)_3Na^+$
622.567	$(NaI)_4Na^+$
772.461	$(NaI)_5Na^+$
922.355	$(NaI)_6Na^+$
1072.250	$(NaI)_7Na^+$
1222.144	$(NaI)_8Na^+$
1372.038	$(NaI)_9Na^+$
1521.932	$(NaI)_{10}Na^+$
1671.827	$(NaI)_{11}Na^+$
1821.721	$(NaI)_{12}Na^+$
1971.615	$(NaI)_{13}Na^+$

Supplementary Table 4. List of equipments used in the t-SPESI measurement system.

Unit name	Name	Model	Manufacturer	Country
Control unit	PXI controller	cRIO-9047	NI	USA
	A/D converter	NI9215	NI	USA
	D/A converter	NI9263	NI	USA
	Relay	NI9482	NI	USA
	Power supply for PXI controller	PS-16	NI	USA
	Lock-in amplifier	LI5650	NF	Japan
	High voltage source for ESI	610C	Trek	USA
	PC (microscope)	Precision 3660	DELL	USA
	PC (t-SPESI)	Precision 3640	DELL	USA
t-SPESI unit	Laser unit	TC-20-DCGS-RP	NEO ARK	Japan
	Piezo actuator	PMF-3020	NTK Ceratek	Japan
	Laser controller	DPS-5004	NEO ARK	Japan
	Probe XYZ stage controller	HIT-S/HIT-M	OptoSigma	Japan
	Probe X stage	SGSP26-50	OptoSigma	Japan
	Probe Y stage	SGSP20-35	OptoSigma	Japan
	Probe Z stage	OSMS20-85	OptoSigma	Japan
	Probe Z piezo stage controller	NCS-7102C	THK Precision	Japan
	Probe Z piezo stage	B22-083	THK Precision	Japan
	Probe vibration piezo amplifier	M-26109-OU	MESS-TEK	Japan
Microscope unit	Optical microscope	TE2000-U	Nikon	Japan
	Microscope stage	BIOS-206T	OptoSigma	Japan
	Microscope stage controller	FC-101G	OptoSigma	Japan
	Light source for fluorescence imaging	C-HGFIE	Nikon	Japan
	Camera	DS-Fi3	Nikon	Japan
	Capture software	NIS-Elements	Nikon	Japan
	USB camera	IC1500CU	Shodensha	Japan
	USB camera	IC1300CU	Shodensha	Japan
	Macro zoom lens	MLH-10X	Computar	Japan
Ion transfer tube	Temperature controller	MTCS	Misumi	Japan
Others	Background reduction	ABIRD	ESI Source Solutions	USA
	Isolation table	HAX-0806	JVI	Japan
	Oscilloscope	DSOX2004A	KEYSIGHT	USA

Supplementary Table 5. Optimized MRM parameters for lipidomic analysis via SFC/MS/MS.

Polarity	Positive	Negative
Sheath gas flow rate	10 arb	50 arb
Aux gas flow rate	0 arb	10 arb
Spray voltage	3.5 kV	-2.0 kV
Ion transfer tube temperature	320 °C	320 °C
S-lens level	60	60
Vaporizer temperature	100 °C	100 °C
Polarity	Positive	Negative
Full MS		
Resolution	60000	60000
AGC target	1×10^6	1×10^6
Maximum IT	200 ms	200 ms
Scan range	200-2000	200-2000

Supplementary Table 6. List of lipids.

Lipid name	Formula	Exact mass	SFC-MS/MS results: Adduct	Measured m/z	RT (min)	GCS amount (amol/cell)	mCAT amount (amol/cell)	Ratio (GCS/mCAT)	t-SPESI results: Measured m/z	Matched m/z	Delta (ppm)	Ion	GCS averaged signal intensity	mCAT averaged signal intensity	Signal intensity ratio (GCS/mCAT)	t-test p-value	Levene's test p-value
PC 30:1	C38H74N1O8P1	703.515205	$[M+H]^+$	704.523005	7.5±0.01	134.448	191.477	0.702	704.527	704.523	5.8	$[M+H]^+$	604.2	870.0	0.69	7.7E-18	5.9E-04
PC 30:0	C38H76N1O8P1	705.530855	$[M+H]^+$	706.538655	7.44±0.02	446.050	734.354	0.607	706.541	706.538	3.4	$[M+H]^+$	2902.3	4094.3	0.71	5.4E-17	2.3E-01
SM d18:1/16:0	C39H79N2O6P1	702.5675749	$[M+H]^+$	703.5753749	8.05±0.02	807.615	1435.566	0.563	725.557	725.557	0.8	$[M+Na]^+$	643.8	971.3	0.66	4.9E-10	9.4E-04
SM d18:1/18:1	C41H81N2O6P1	728.583225	$[M+H]^+$	729.591025	8.11±0.02	39.604	73.805	0.537	729.587	729.591	4.3	$[M+H]^+$	351.4	311.5	1.13	9.1E-07	6.7E-02
PC 32:2	C40H76N1O8P1	729.530855	$[M+H]^+$	730.538655	7.53±0.02	366.988	475.550	0.772	730.537	730.538	1.7	$[M+H]^+$	1089.6	1690.6	0.64	1.6E-19	1.6E-05
PC 32:1	C40H78N1O8P1	731.5465051	$[M+H]^+$	732.5543051	7.47±0.01	1779.388	2645.875	0.673	732.555	732.554	1.7	$[M+H]^+$	8249.8	10408.1	0.79	3.5E-10	6.0E-02
PC 32:0	C40H80N1O8P1	733.5621552	$[M+H]^+$	734.5699552	7.42±0.02	580.219	1012.155	0.573	734.570	734.569	0.2	$[M+H]^+$	9691.3	12093.8	0.80	4.9E-07	4.9E-01
SM d18:1/16:0	C39H79N2O6P1	702.5675749	$[M+H]^+$	703.5753749	8.05±0.02	807.615	1435.566	0.563	741.532	741.531	1.9	$[M+K]^+$	2063.1	1443.1	1.43	9.7E-09	1.5E-03
PE 36:2	C41H78N1O8P1	743.5465051	$[M+H]^+$	744.5543051	9.17±0.02	18077.309	27183.895	0.665	744.558	744.554	5.5	$[M+H]^+$	1287.9	1041.6	1.24	7.0E-06	1.2E-04
SM d18:1/18:2	C41H79N2O6P1	726.5675749	$[M+H]^+$	727.5753749	8.14±0.02	0.881	1.674	0.526	749.560	749.557	4.0	$[M+Na]^+$	912.4	1410.0	0.65	1.2E-08	1.4E-05
PE 34:0	C39H78N1O8P1	719.5465051	$[M+H]^+$	720.5543051	9.12±0.02	560.148	795.620	0.704	758.505	758.510	6.4	$[M+K]^+$	1599.2	1175.4	1.36	1.7E-08	3.3E-02
PC 34:2	C42H80N1O8P1	757.5621552	$[M+H]^+$	758.5699552	7.51±0.01	1335.567	2087.701	0.640	758.569	758.569	1.2	$[M+H]^+$	4242.8	7254.5	0.58	7.9E-24	2.1E-02
PC 34:1	C42H82N1O8P1	759.5778052	$[M+H]^+$	760.5856052	7.44±0.01	1993.658	3464.749	0.575	760.585	760.585	0.2	$[M+H]^+$	19057.5	21417.0	0.89	4.8E-04	6.1E-01
PC 32:3	C40H74N1O8P1	727.515205	$[M+H]^+$	728.523005	7.54±0.01	9.312	13.949	0.668	766.479	766.478	0.6	$[M+K]^+$	464.7	400.5	1.16	1.4E-08	1.4E-01
SM d18:1/18:0	C41H83N2O6P1	730.598875	$[M+H]^+$	731.606675	8.03±0.02	113.219	197.347	0.574	769.559	769.562	3.5	$[M+K]^+$	1542.9	1203.5	1.28	1.9E-06	6.6E-05
PC 34:0	C42H84N1O8P1	761.5934553	$[M+H]^+$	762.6012553	7.41±0.03	136.404	231.174	0.590	784.578	784.583	5.9	$[M+Na]^+$	3083.2	3858.3	0.80	9.5E-09	2.6E-01
HexCer d18:1/22:0	C46H89N1O8	783.6588188	$[M+H]^+$	784.6666188	7.63±0.01	33.231	2.680	12.400	784.667	784.666	1.7	$[M+H]^+$	473.1	297.8	1.59	1.7E-12	2.8E-13
PC 36:2	C44H84N1O8P1	785.5934553	$[M+H]^+$	786.6012553	7.48±0.01	1160.222	2147.009	0.540	786.600	786.601	0.5	$[M+H]^+$	5879.7	6798.1	0.86	2.0E-03	1.4E-01
SM d18:1/22:0	C45H91N2O6P1	786.6614753	$[M+H]^+$	787.6692753	7.97±0.02	106.753	291.424	0.366	787.669	787.669	0.4	$[M+H]^+$	779.4	1555.4	0.50	3.6E-25	4.5E-01
PC 36:1	C44H86N1O8P1	787.6091053	$[M+H]^+$	788.6169053	7.41±0.02	132.987	271.316	0.490	788.613	788.616	3.8	$[M+H]^+$	3722.5	2929.7	1.27	2.6E-08	8.5E-01
PC 36:2	C44H84N1O8P1	785.5934553	$[M+H]^+$	786.6012553	7.48±0.01	1160.222	2147.009	0.540	808.580	808.583	3.5	$[M+Na]^+$	3396.6	5078.1	0.67	3.0E-17	2.3E-02
SM d18:1/22:0	C45H91N2O6P1	786.6614753	$[M+H]^+$	787.6692753	7.97±0.02	106.753	291.424	0.366	809.647	809.651	4.1	$[M+Na]^+$	513.3	846.6	0.61	2.6E-17	7.0E-05
PS 38:1	C44H84N1O10P1	817.5832845	[M-H]-	816.5754845	17.46±0.04	48.338	54.857	0.881	818.590	818.591	1.1	$[M+H]^+$	1485.2	1901.2	0.78	3.1E-05	9.8E-10
SM d18:1/24:0	C47H95N2O6P1	814.6927754	$[M+H]^+$	815.7005754	7.95±0.02	66.894	221.045	0.303	837.680	837.682	2.9	$[M+Na]^+$	370.8	547.1	0.68	5.7E-16	1.9E-07
PC 38:4	C46H84N1O8P1	809.5934553	$[M+H]^+$	810.6012553	7.53±0.03	116.267	211.815	0.549	848.556	848.557	0.8	$[M+K]^+$	3182.7	1620.7	1.96	2.0E-15	2.4E-06
PS 40:3	C46H84N1O10P1	841.5832845	$[M-H]^-$	840.5754845	17.58±0.03	22.918	32.080	0.714	880.549	880.546	2.8	$[M+K]^+$	1152.2	755.2	1.53	2.5E-14	3.5E-03
TG 52:9	C55H88O6	844.6580905	$[M+NH4]^+$	862.6924396	4.1±0.02	0.250	0.293	0.853	883.624	883.621	3.3	$[M+K]^+$	484.2	595.2	0.81	2.5E-10	3.1E-07
PC 42:9	C50H82N1O8P1	855.5778052	$[M+H]^+$	856.5856052	7.75±0.02	2.349	4.022	0.584	894.537	894.541	4.7	$[M+K]^+$	893.6	684.0	1.31	2.1E-13	2.4E-01
PS 42:5	C48H84N1O10P1	865.5832845	[M-H]-	864.5754845	17±0.02	397.555	469.256	0.847	904.547	904.546	0.2	$[M+K]^+$	712.5	656.7	1.09	2.9E-03	9.2E-02
